# Retraction: Synthesis of metal nanoparticles on graphene oxide and antibacterial properties

**DOI:** 10.3389/fchem.2026.1816414

**Published:** 2026-03-04

**Authors:** Frontiers Editorial Office

Following publication, concerns were raised regarding similarities in data content between this paper and a manuscript published prior by the same authors’ group: Tene, T. et al. A Facile and Green Approach for the Preparation of Silver Nanoparticles on Graphene Oxide with Favorable Antibacterial Activity. Nanomaterials 2024, 14, 1455. https://doi.org/10.3390/nano14171455

Given the extent of the content overlap, the article does not meet the standards of editorial and scientific rigor for Frontiers in Chemistry; therefore, the article has been retracted.

This retraction was approved by Chief Editor of Frontiers in Chemistry and the Chief Executive Editor of Frontiers. The authors did not agree with this retraction. Frontiers would like to thank Dr. Abbas Aziz for contacting the journal regarding the published article.

